# Active tuberculosis case findings in Ghanaian health facilities: effectiveness and sensitivity of the symptoms-based screening tool

**DOI:** 10.11604/pamj.2021.40.111.28798

**Published:** 2021-10-20

**Authors:** Kwabena Obeng Duedu, Enoch Aninagyei, Diana Ayinpokbila Akila, Margaret Kweku

**Affiliations:** 1 Department of Biomedical Sciences, School of Basic and Biomedical Sciences, University of Health and Allied Sciences, Hohoe, Ghana,; 2 Department of Epidemiology and Biostatistics, School of Public Health, University of Health and Allied Sciences, Hohoe, Ghana

**Keywords:** Tuberculosis, active case finding, hospital, symptoms-based screening tool

## Abstract

**Introduction:**

the National Tuberculosis Programme (NTP), Ghana, introduced Symptoms-Based Screening (SBS) Tool for TB case finding. This study aimed to determine the challenges and limitations associated with the use of the SBS Tool for active tuberculosis case finding in Ghanaian health facility settings.

**Methods:**

this study targeted suspected TB patients attending two health facilities in the Ho Municipality, Ghana. Initially, suspected TB patients were screened with the SBS tool and presumptive patients subsequently tested for M. tuberculosis using microscopy and geneXpert assay. Additionally, health personnel were interviewed to assess the user-friendliness, challenges, and limitations associated with the tool.

**Results:**

of 636 presumptive TB patients identified, 1.73% had tuberculosis. Coughing for > 2 weeks (χ^2^=24.8; p<0.05); chest pain (χ^2^=28.3; p<0.01) and night sweat (χ^2^=34.8; p<0.05) associated significantly with M. tuberculosis infection status. The health personnel found the tool to be not user-friendly and it also lacked indicators to identify other vulnerable individuals such as diabetics, cigarette smokers, alcoholics, immunocompromised, and malnourished individuals. Therefore, the SBS tool was found not to be sensitive enough to identify probable cases.

**Conclusion:**

the SBS tool is useful for detecting active TB cases, however, it must be improved to identify vulnerable individuals such as diabetics, immunosuppressed, and malnourished.

## Introduction

There are two outcomes of *Mycobacterium tuberculosis* (MTB) infection; latent and active. Many people exposed to MTB bacilli are immunocompetent and remain asymptomatic [1, 2]. Several factors make people more susceptible to TB infections; people living with HIV/AIDS, chronic lung disease, smoking cigarettes, excessive drinking of alcohol, and diabetics [3, 4]. Tuberculosis is also linked to overcrowding and malnutrition and among the group of people with limited movement like prisoners [5]. Students in hostels are also at a higher risk of infection. Again, resource poor community dwellers and children who are in close contact with infected patients are also at risk [6, 7].

In latent TB, the bacteria remain in the body in an inactive state. There are no symptoms associated with this, and the affected individuals are not contagious. This can however transform to an active form of the disease. About one-third of the world's population is believed to have latent TB. About 10% become active at a time in their lives, especially in people with compromised immunity [8].

TB remains a major public health problem in Ghana despite the progress made in combating the disease over the past years. In 2020, the estimated national prevalence of TB in Ghana by microscopy was 111 per 100,000 population while bacteriologically confirmed TB was 356 per 100,000 population [9]. This recently reported prevalence could be under-reported since most out-patient department (OPD) is Ghanaian health facilities do not actively search for TB cases. Based on this, the National Tuberculosis Control Programme, Ghana developed a paper-based screening tool called the Symptom-Based Screening (SBS) Tool (Chest Infection) for TB active case findings. There is no hospital-based report on the sensitivity and effectiveness of the SBS Tool in the Volta Region of Ghana. However, a community-based study in the region reported prevalence of 15/100,000 [10]. Despite the progress made in combating TB over the past years, there is still a gap in TB case detection in the country and in the Volta Region of Ghana.

This study was implemented in two health facilities in the southern part of Volta Region, Ghana, to determine the effectiveness and sensitivity associated with the SBS Tool for TB active case findings. Specifically, this study determined the prevalence of TB among patients presenting at the OPDs of these two hospitals following initial screening with the SBS Tool and determined the challenges faced by practitioners and factors limiting the sensitivity of the screening tool.

## Methods

**Study design, study sites, and period of sample collection:** this study was a health facility-based cross-sectional study that occurred from December 2018 to January 2019 in Ho Teaching and Ho Municipal hospitals in the Volta Region, Ghana. The Symptom-Based Screening Tool (SBS) for TB active case findings was administered at each consulting room in the hospital. From patients suspected of TB, two sputum samples were collected for the determination of *M. tuberculosis* DNA at the Regional Hospital Laboratory in Ho.

**Study site description:** the study was carried out in the southern part of the Volta Region of Ghana. Then Volta region was divided into 25 administrative districts (now Oti Region has been carved out of the Volta region). Ho is the capital of the Ho Municipality and the Volta Regional capital. Ho is a cosmopolitan city with brisk economic, social activities as well as several tourist attractions. Again, the municipality has several second cycle instruction, nursing training colleges, colleges of education, technical university, and health and allied sciences university as well as several private universities. Moreover, there is a big market and other small markets in the municipality. This attracts a lot of people into the municipality and overcrowding is a frequent occurrence. The two biggest hospitals in the Municipality were Ho Teaching Hospital (HTH) and Ho Municipal Hospital (HMH).

**Training of data collection team:** nurses were trained at each hospital as data collectors. Ten (10) nurses were trained at each health facility for data collection. Pre-testing of the survey tools was done during the training period in the Ho Municipal hospital. This afforded the team the opportunity to have practical experience with the administration of the study tools.

**Inclusion and exclusion criteria:** individuals aged 15 years and above were eligible for inclusion. Additionally, patients that have a history of cough for two weeks or more were included in the study. Persons excluded from the study were seriously ill patients who could not provide informed consent as well as those that could not produce sputum. All other presumptive tuberculosis patients that did not fall within the study criteria were managed routinely by existing tuberculosis management protocols in Ghana. Written consent was obtained from the study participants.

**Sample size estimation:** the sample size for this study was calculated using the formulae below:


$$N=\frac{z^{2}P(1-P)}{e^{2}}$$

Where: N = calculated sample size, Z = Z score (reliability coefficient) of 1.96 at 95% confidence interval, P = Prevalence of 0.282 which was the prevalence of TB per 100,000 population in the Volta region for the year 2017 (Volta Regional Health Directorate, 2017) and e = margin of error (0.05). Using the above formula, the sample size was calculated to be 311. Adjusting for a 5% nonresponse rate, the minimum sample size of 327 was used for this study.

**Sampling of participants:** patients who met the inclusion criteria were recruited into the study at both the OPD and in the consulting rooms of each health facility. All patients who visited the two hospitals during the study period and consented to participate in the study were sampled. Those who consented to participate were screened at a designated place at the OPD to ensure privacy and confidentiality.

**Administration of SBS tool:** the Symptom-based screening (SBS) tool (adapted from an existing National Tuberculosis Control Program screening tool) was administered to suspected TB patients who satisfied the inclusion criteria. The SBS tool was administered to individuals who complained of coughing for two weeks or more. Other variables were collected: age, sex, chest pain, weight lost, night sweat, and fever. Anyone who met the aforementioned criteria was classified as patients with presumptive TB and sputum was collected from them for examination for *M. tuberculosis*. Additionally, health workers who manage TB patients were also interviewed on the feasibility of the SBS tool.

**Assessment of challenges and limitations of the SBS tool:** twenty-four health personnel of different cadres were randomly selected from each of the two health facilities. The health personnel were interviewed to assess the user-friendliness, efficiency, challenges, and limitations associated with the SBS tool. A semi structured questionnaire was used to elicit responses.

**Sputum collection for TB diagnosis:** as recommended by National Tuberculosis Control Programme, two sputum samples were collected from each presumptive *M. tuberculosis* individual; a spot sample and a second sample after one hour. Presumptive *M. tuberculosis* individuals were instructed on how to induce sputum production. They were made to cough three times after taking a deep breath to produce sputum. Sputum samples were kept in tight containers and transported to the laboratory within 24 hours for microbiological examination. Each sample pair was examined using Ziehl Neelsen microscopy technique and MTB/RIF geneXpert analyser.

**Procedure for HIV screening:** all eligible participants who agreed to be tested for HIV were referred to the HIV Counselling and Testing Unit of the hospital for counselling before being tested for HIV using a rapid diagnostic test (RDT) (First response HIV1&2 kit, Premier Medical Corporation Ltd., Kachigam, India).

**Data analysis:** study data were recorded on Microsoft Excel spreadsheet (2016) and were subsequently verified for inconsistency, completeness, and accuracy. Data were then exported to STATA version 14.1 for analysis. Simple frequencies and percentages were used for categorical variables while Chi-square was used to determine the association between the dependent (prevalence of pulmonary TB) and independent variables p-value <0.05 was considered statistically significant.

## Results

**Demographic characteristics of presumptive people with *M. tuberculosis*:** a total of 636 presumptive people with *M. tuberculosis* were sampled from Ho Teaching Hospital (HTH) and Ho Municipal Hospital (HMH). Majority of presumptive cases in each demographic indicator was females and patients above 60 years. Christians were in the majority, so as married individuals. Frequencies of these and other parameters are presented in Table 1. Table 2 represents the demographic profile of the health workers who consented to answer the study questionnaires. In all, 24 health care workers provided responses to the study questionnaire. Most of the workers in both facilities were aged 23-33 years. Whereas most of the staff were married in HMH, single staff dominated in VHR. Almost all staff have had tertiary education, with the majority of them being Ewes. The dominant cadre of staff that were interviewed were nurses.

**Table 1:** demographic characteristics of presumptive people with *M. tuberculosis*

Health facilities (n=636)			
	HTH (n = 323)	HMH (n = 313)	Total (N=636)
	n (%)	n (%)	n (%)
**Sex**			
Male	117 (36.2)	88 (28.1)	205 (32.2)
Female	206 (63.8)	225 (71.9)	431 (67.8)
**Age group in years**			
15 – 29	90 (27.9)	70 (22.4)	160 (25.2)
30 – 44	66 (20.4)	80 (25.5)	146 (22.9)
45 – 59	60 (18.6)	76 (24.3)	136 (21.4)
> 60	107 (33.1)	87 (27.8)	194 (30.5)
**Religion**			
Christianity	315 (97.5)	301 (96.2)	616 (96.8)
Islamic	8 (2.5)	9 (2.8)	17 (2.7)
Traditionalist	0 (0)	3 (1.0)	3 (0.5)
**Tribe**			
Ewe	303 (93.8)	295 (94.2)	598 (94.0)
Guan	14 (1.6)	6 (1.9)	20 (3.2)
Akan	4 (1.2)	4 (1.3)	8 (1.3)
Ga	2 (0.6)	8 (2.6)	10 (1.6)
**Marital Status**			
Single	110 (34.1)	71 (22.7)	181 (28.5)
Married	171 (52.9)	216 (69.0)	387 (60.8)
Divorced	6 (1.9)	2 (0.6)	8 (1.3)
Widowed	36 (11.1)	24 (7.7)	60 (9.4)
**Educational Level**			
None	58 (18.0)	57 (18.2)	115 (18.1)
Basic	92 (28.5)	86 (27.5)	178 (28.0)
Secondary	106 (32.8)	107 (34.2)	213 (33.5)
Tertiary	67 (20.7)	63 (20.1)	130 (20.4)
**Department**			
Consulting Room	121 (37.5)	143 (45.7)	264 (41.5)
OPD Front Desk	202 (62.5)	170 (54.3)	372 (58.5)


HTH = Ho Teaching Hospital; HMH = Ho Municipal Hospital; 1 others: figures are expressed as number (%)

**Table 2:** demographic characteristics of health workers

	Health facilities (n=24)		
Demographic indicators	HMH (n=14)	HTH (n=10)	Total (n=24)
	n (%)	n (%)	n (%)
**Sex**			
Male	5 (35.7)	7 (70.0)	12 (50.0)
Female	9 (64.3)	3 (30.0)	12 (50.0)
**Age group in years**			
23-33	10 (71.4)	7 (70.0)	17 (70.8)
43-43	2 (14.3)	3 (30.0)	5 (20.8)
44+	2 (14.3)	0 (0)	2 (8.4)
**Marital Status**			
Single	5 (35.7)	8 (80.0)	13 (54.2)
Married	9 (64.3)	2 (20.0)	11 (45.8)
**Religion**			
Christianity	14 (100.0)	9 (90.0)	23 (95.8)
Islam	0 (0)	1 (10.0)	1 (4.2)
Tribe			
Ewe	11 (78.6)	8 (80.0)	19 (79.2)
Guan	0 (0)	1 (10.0)	1 (4.2)
Akan	3 (21.4)	1 (10.0)	4 (16.6)
**Educational Level**			
Secondary	1 (7.1)	0 (0)	1 (4.2)
Tertiary	13 (92.9)	10 (100.0)	23 (95.8)
**Cadre of staff**			
Medical Officers	2 (14.2)	4 (40.0)	6 (25.0)
Nurses	6 (42.9)	6 (60.0)	12 (50.0)
Physician assistants	6 (42.9)	0 (0)	6 (25.0)


HTH = Ho Teaching Hospital; HMH = Ho Municipal Hospital, figures are expressed as number (%)

**Prevalence of *M. tuberculosis* and sensitivity of the TB symptom-based tool:** in all, 4 and 7 positive cases were recorded in HTH and HMH, respectively, representing 1.24% and 2.17% HTH and HMH, respectively. The overall prevalence of tuberculosis found in the two health facilities was 1.73%. The overall sensitivity of the SBS tool in detecting *M. tuberculosis* was 1.7%, while the hospital-related sensitivities were 1.2% and 2.2% in HTH and HMH, respectively.

**Figure 1:**
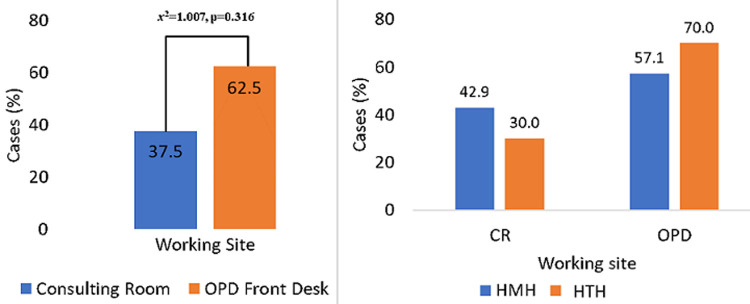
departmental and health facility specific for the administration of the SBS tool for *MTB* screening by Health Workers (A) overall preferred site for the administration of the tool; (B) distribution of preferences within the respective health facilities studied; Key: HTH = Ho Teaching Hospital; HMH = Ho Municipal Hospital; CR = Consulting Room; OPD = Out Patient Department Front Desk; SBS-symptom-based screening

**Preferred department of administration of TB symptom-based tools:** the preferred location of screening for *M. tuberculosis* using SBS was the OPD Front Desk. However, the difference in the cases recruited from OPD and consulting rooms was not significant (χ^2^=1.007, p=0.316). Similar observation was made for each of the 2 health facilities (Figure 1).

**Association between *M. tuberculosis* infection status and symptoms:** there was a statistically significant association between TB infection status and cough more than 2 weeks (χ^2^=24.8; p<0.001); chest pain and *M. tuberculosis* status (χ^2^=28.3; p<0.001) and night sweat and *M. tuberculosis* infection status (χ^2^=34.8; p<0.001 (Table 3).

**Table 3:** chi-square association between *M. tuberculosis* infection status and presenting symptoms

	TB Status				
Variable	Positive (n=11)	Negative (n=625)	Total (n=636)	Chi-square	p-value
**Cough > 2 weeks**					
Yes	9 (81.8)	125 (20.0)	134 (21.1)		
No	2 (18.2)	500 (80.0)	502 (78.9)	24.8	<0.001
**Chest pain**					
Yes	11 (100.0)	169 (27.0)	180 (28.3)		
No	0 (0)	456 (73.0)	456 (71.7)	28.4	<0.001
**Night sweat**					
Yes	7 (63.6)	58 (9.3)	65 (10.2)		
No	4 (36.4)	567 (90.7)	571 (89.8)	34.8	<0.001
**Department**					
CR	7 (63.6)	257 (41.1)	264 (41.5)		
OPD	4 (36.4)	368 (58.9)	372 (58.5)	2.3	0.133


Figures are expressed as number (%); CR-consulting room; OPD-out-patient department

**Implementation challenges associated with the SBS tool:** of the total staffs, 15 (62.5%) did not have any challenges administering the tool, while 9 of them had some challenges with the tool. The common challenges identified were inadequate health staff, lack of patient´s cooperation to provide responses, font size being too small, too many questions, and filling hard copies of several questions is cumbersome and boring.

**Limitations of the SBS tool:** inability of the tool to capture other risk groups such as diabetics, cigarette smokers, alcoholics, immunocompromised, and malnourished individuals was reported by the health care providers as the main limitation of the tool.

## Discussion

This current study screened 636 people with TB out of which 1.73% (1730 per 100 000 population) infected with *M. tuberculosis*. A similar study in Ethiopia reported a higher prevalence (4.4%) of identified TB cases [11]. Similarly, in Tanzania, 4.1% of TB were detected in people with TB screened with the Symptom-Based Screening Tool [12]. The differences in the prevalence reported in these studies could be as a result of the differences in the study population in each study and the time the sputum sample was collected. In this study, none of the presumptive patients was either HIV positive or immunocompromised. Additionally, sputum samples were collected from presumptive TB patients at any time of day, meanwhile, a previous study has reported that an early morning sputum sample is necessary for TB detection, even with the most sensitive diagnostic technique [13]. Early morning sample is clearly ideal for MTB detection due to the concentration of the bacilli overnight with possible high number of them in an expectorating sputum.

In the study reported by Menberu *et al*. [11] and Shayo *et al*. [12]. The screening tool was administered to HIV-infected persons with TB. Therefore, it was not surprising that they had a relatively high prevalence of TB among the participants. HIV infections lead to host immunosuppression and thereby rendering the host susceptible to *M. tuberculosis* infection. Again, HIV is the most important risk factor for tuberculosis [14]. In contrast to this study, presumptive people with TB were negative for HIV based on the rapid testing kit. However, the findings in this study underscore the fact that the infected cases could have been missed with the implementation of the SBS tool. A lower prevalence was reported in our study could be because the study was carried out in an urban population, with its attendant low prevalence of factors that cause immunosuppression. Chest X-ray has also been used to identify individuals with MTB when microscopy fails to detect bacilli in sputum, but this technique has been found to be inferior to GeneXpert assay, therefore Nakiyingi *et al*. established that chest X-ray interpretation may not add diagnostic value in settings where Xpert MTB/RIF is available as a TB diagnostic tool [15].

Considering the socio-economic nature of Ghana, communal living and communal activities are widespread. If the infected individuals are not treated promptly, they will remain a continuous high-risk source of infection to their contacts. As soon as people with TB begin standard TB medication, their infectiousness reduces. Previous studies found that after anti-TB chemotherapy, *M. tuberculosis* bacilli burden is reduced to approximately 4% of the initial bacilli load within 48 hours after the start of treatment and to 1% within 2-3 weeks of anti-TB chemotherapy in drug-sensitive TB patients [16, 17].

Detection of only 1.73% of the presumptive TB cases reveals the lack of sensitivity of the screening tool even though geneXpert is very sensitive and specific for MTB. However, analysis of data obtained in this study concluded that there were a strong association between cough more than 2 weeks, chest pain, and night sweat, and TB infection status. It is imperative that clinicians´ survey for these symptoms, irrespective of the clinical complains and get them tested for tuberculosis using geneXpert.

Few operational limitations to the use of SBS tools were identified in this study. The health workers indicated that the screening tool is laborious to administer. Likewise, Reid and Shah [18] also reported that a similar screening tool available to them was not user-friendly. Another major limitation identified by the health providers was the inability of the tool to capture other risk groups such as individuals with chronic lung disease, cigarette smokers, chronic alcoholics, diabetics, pregnant women in their 2^nd^ trimester, prisoners, individuals with prolonged stay in overcrowded environments (market women, students in hostels etc.) as well as malnourished individuals. Moreover, the tool did not allow for screening of people who live in resource poor communities like slums. These individuals have been found to be at higher risk of tuberculosis disease [19].

## Conclusion

This study was able to identify some patients infected with TB in the study area. This underscores the fact that active TB case search is an essential exercise in identifying cases that would have hitherto been lost. Such an individual poses a threat to public health and disrupts national program efforts. Even though the current SBS tool detected 1.73% of asymptomatic TB, it could be simplified to make it user-friendly and redesigned to capture other high-risk groups.

### What is known about this topic

- Mycobacterium tuberculosis causes tuberculosis;
- Several people have tuberculosis but escape undetected due to low bacilli load and presentation of non-specific symptoms;
- National Tuberculosis Control Programme, Ghana introduced a symptom-based screening tool to increase the detection of active tuberculosis.

### What this study adds

- The symptom-based screening tool is able to identify up to 1.73% of individuals infected with Mycobacterium tuberculosis;
- The tool is not user-friendly to health workers that use it. However, in both health facilities, personnel stationed at the outpatient department (OPD) were familiar with the tool.
- One point two percent (1.2%) of presumptive TB patients sought care at the Ho Teaching Hospital while that of Ho Municipal Hospital was 2.2%.
